# Floristic analyses of Shandong peninsula and adjacent areas indicate the barrier effect of the Yellow river on floristic diversity

**DOI:** 10.3389/fpls.2024.1419876

**Published:** 2024-08-15

**Authors:** Suyin Wang, Qing Xian, Liu Yang, Wei Zhang

**Affiliations:** Marine College, Shandong University, Weihai, Shandong, China

**Keywords:** flora of China, floristic composition, phytogeography, plant diversity, riverine barriers, Shandong

## Abstract

The Shandong Peninsula, the largest peninsula in China, is situated at the estuary of the Yellow River and is bordered by both the Bohai Sea and the Yellow Sea. This region is renowned for its rich plant diversity. However, the historical origins of these plant species remain poorly understood. This study analyzed 2410 shared species from 865 genera and 161 families distributed across Shandong and its nine adjacent regions to investigate the floristic diversity of the Shandong Peninsula. These regions were considered as operational taxonomic units (OTUs), with the shared species serving as the basis for each OTU. Hierarchical cluster analyses were performed to assess their floristic similarity, employing the Bray-Curtis distance algorithm and the UPGMA clustering method. The results revealed that the ten regions were grouped into three clusters, delineated by the Yellow River. Notably, the floristic similarity of the Shandong Peninsula was found to be more closely aligned with regions south of the Yellow River, despite Shandong historical connection to Liaoning in the north. These findings underscore the barrier effect of the Yellow River and provide insights into the formation of biotic diversity patterns between northern and eastern China.

## 1 Introduction

China, which boasts an impressive array of approximately 30,000 species of seed plants (Wu et al., 2013), is one of the countries with the highest global species diversity. This remarkable diversity, apart from the relation with the wide extension of the country, arises primarily from China’s geographical variety, encompassing mountains, plains, and hills (Yang et al., 2005). Additionally, the country intricate water systems, including rivers, lakes, and other natural water bodies, further contribute this diversity. These diverse habitats serve as cradle for rapid speciation, fostering the evolution and adaptation of novel species to distinct environments. Similarly, they act as museum of ancient species, maintaining lineages across extensive geological timescales (Li, 2008; Lu et al., 2018).

Over the years, researchers in China have concentrated on studying species diversity and geographical distribution. Wu developed a 15-areal type classification framework for seed plant genera distribution patterns based on the distribution of around 3000 genera (Wu, 1979, 1991; Wu et al., 2010). This scheme has been extensively employed for analyzing national and regional floras of China at various levels, fostering a surge of research on numerous regional floras and leading to the publication of numerous focused works (Sun et al., 2017). The integration of molecular data and quantitative methodologies has driven remarkable advancements in China’s phytogeography (e.g. Liu et al., 2012; Zhang et al., 2016; Lu et al., 2018). Recently, Ye et al. (2019) used a phylogenetic dissimilarity approach to regionalize the Chinese flora into 943 distinct grid cells, organizing them into five phytogeographical regions: Paleotropic, Holarctic, East Asiatic, Tethyan, and Qinghai–Tibet Plateau.

However, the current phytogeographical classification of China remains framework-based, primarily relying on endemism and its hierarchical levels. This includes using endemic families to divide the realm or kingdom and endemic genera to delineate regions (Sun et al., 2017; Ye et al., 2019). Due to the lack of detailed geographical data and species-level analysis in early stages, these biogeographical classifications are hindered from undergoing deeper analysis and finer categorization (Li, 2008). Given China intricate geography and varying climatic conditions, there remains a substantial gap in our understanding of species diversity and its underlying mechanisms within numerous small, habitat-specific regions.

**Figure 1:**
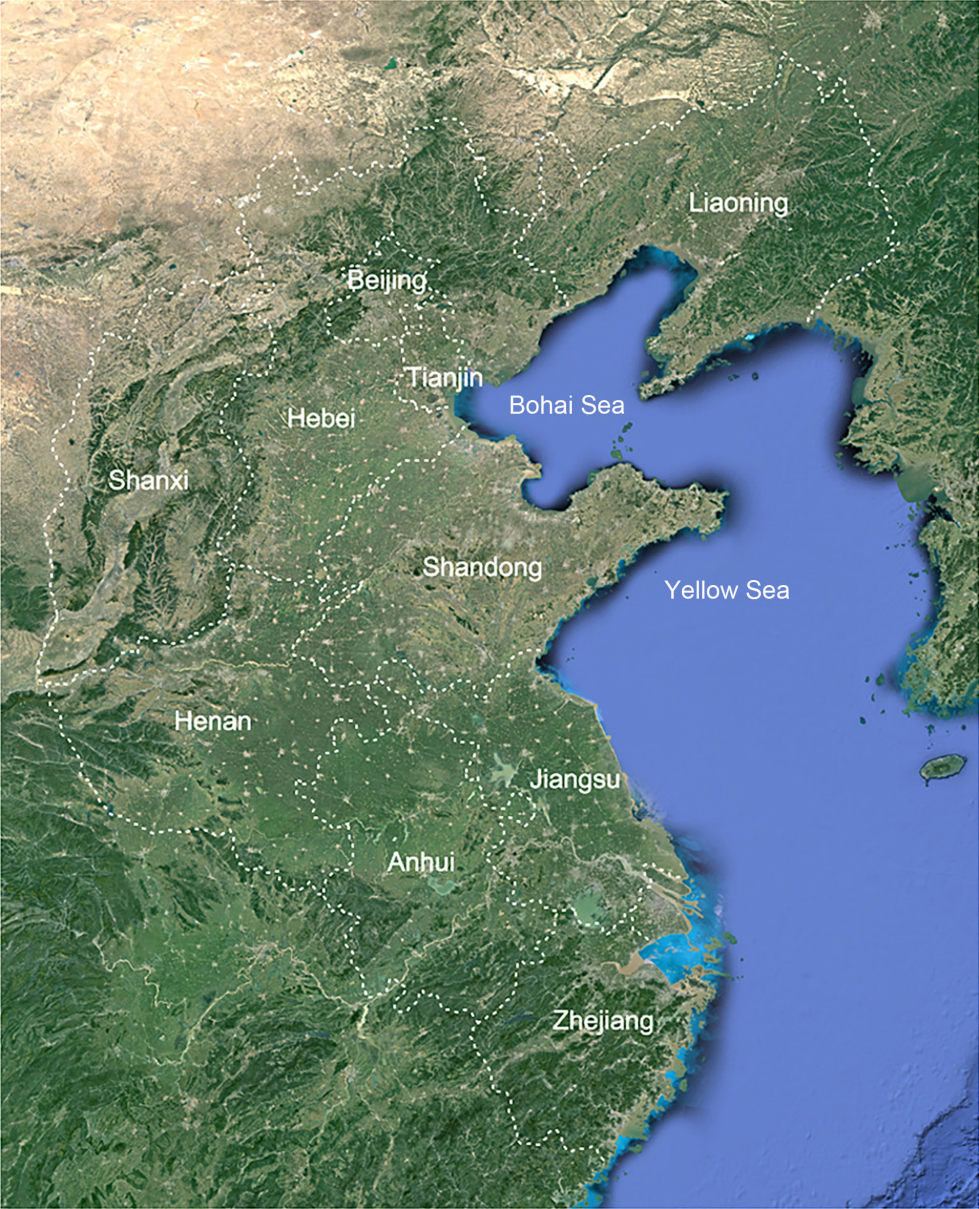
Location map of the study area of Shandong Peninsula and adjacent areas.

The Shandong Peninsula, the largest in China, is situated between the Bohai Sea and Yellow Sea, with the Yellow River flowing through its northern region. It borders the Liaodong Peninsula and Japan to the north, while facing South Korea to the east (**Figure 1**). During the Tertiary Period, the Shandong Peninsula was geologically connected to the Liaodong Peninsula and resided within the northern subtropical region (Jin and Yu, 1982). This geological connection facilitated the merging of tropical flora from the south with the Changbai Mountain flora from the north. For instance, numerous subtropical plants, such as *Camellia japonica* L. and *Machilus thunbergii* Siebold & Zucc., have migrated northward, reaching their northernmost limit in this region. Similarly, plants indigenous to the Northeastern regions, including *Maackia amurensis* Rupr. & Maxim. and *Clematis patens* C. Morren & Decne., have extended their range southward, achieving their southernmost limit in the Shandong Peninsula. Consequently, the flora of this region exhibits distinct transitional characteristics, reflecting its unique geological history and ecological position.

To conserve and sustainably utilize the coastal ecosystem of the Shandong Peninsula, it is imperative to acquire a comprehensive understanding of the plant types and characteristics in this region. This study aims to analyze the transitional patterns in species composition observed on the Shandong Peninsula. By gathering a diverse range of species data from Shandong province and its neighboring regions, spanning from Liaoning to Zhejiang, we have minimized the potential for errors that might arise from limited geographical coverage (Soria-Auza and Kessler, 2008). Additionally, the adoption of treating these species as operational taxonomic units (OTUs) in our cluster analysis, a method advocated by Kreft and Jetz (2010), enhances the objectivity in inferring phytogeographical regionalization patterns. These methodologies allows us to draw more precise conclusions about the species composition and transitional patterns on the Shandong Peninsula, laying the foundation for sustainable conservation strategies.

## 2 Materials and methods

### 2.1 Study area

The Shandong Peninsula, the largest peninsula in China, is located in the eastern region of the country, spanning latitudes from 35°05′N to 37°50′N and longitudes from 119°16′E to 122°42′E. It is situated between the Bohai Sea and Yellow Sea, with the yellow River flowing through its northern region. The peninsula faces the Liaodong Peninsula across the Bohai Bay and is adjacent to South Korea to the east (**Figure 1**). The region experiences a temperate monsoon climate, influenced by alternating northern and southern monsoons. This unique topography and climate have resulted in rich biodiversity, featuring plants from both northern and southern China, making it a crucial area for plant distribution at the intersection of these regions. An extensive investigation was conducted into ten neighboring regions, each with distinct climatic conditions and species profiles: Liaoning, Hebei, Beijing, Tianjin, Shanxi, Shandong, Henan, Anhui, Jiangsu, and Zhejiang (**Figure 1**).

### 2.2 Species and their distribution data

To gather comprehensive data on seed plants within the study areas, the plant list provided by the *Flora of China* (Wu et al., 2013) was utilized. This reference enables the recording of total of 3135 seed plant species. To assess differences in plant composition between Shandong Province and neighboring regions, and to streamline the analysis, endemic and widely distributed species (those distributed in ten areas) were excluded from the initial dataset. Additionally, recently cultivated plants and invasive species, due to their non-natural origin and potential to introduce complexities into the analysis, were also excluded. Following these criteria, 2410 species, belonging to 865 genera, 161 families, and 48 orders, were selected for further analysis (**Supplementary Table 1**). To investigate the relationship between species distribution and their ecological adaptation, the selected species were categorized based on their life forms and leaf traits, following the methodology of Diaz et al. (2016). Life forms refer to morphological types such as arbor, shrub, herb, or liana, along with plant posture types like erect, tufted, or rosette. Leaf traits focus on characteristics such as leaf texture, which can be membranaceous, chartaceous, or leathery. These features are detailed in the species description section of the *Flora of China*. For plants that exhibit varying forms throughout their life cycle, the form they display during the mature period was used as the primary recorded feature.

### 2.3 Statistics and analysis

To assess the relative abundance of species within each of the ten provinces, a quantitative approach was adopted. Specifically, the proportion of counties in which a species occurred was calculated to the total number of counties within each province. This methodology was consistently applied to determine the relative abundance of individual genera and families (Chi et al., 2020). To evaluate the ecological adaptability of species composition across various regions, common species were excluded, and the remaining species, along with their life forms and distinct leaf traits, were documented according to the descriptions in the *Flora of China* across all ten designated regions. For better visualization, these data were transformed logarithmically and represented in the form of a heatmap. Additionally, common species across various provincial regions were identified and visually represented using Venn diagram plots. Within the numerical taxonomy framework, the provincial regions were designated as OTUs, with species serving as the informational basis for each OTU. Similarity among the regions was computed based on the presence or absence of specific species in each area. Hierarchical cluster analyses were then performed to further assess floral affinities across different regions, utilizing the methodology proposed by Kreft and Jetz (2010). These analyses employed the Bray-Curtis distance algorithm and the UPGMA clustering method, leveraging the capabilities of the online bioinformatics platform at http://www.ehbio.com/. The species composition across all ten regions was graphically represented using bar plots generated by the ehbio platform at http://www.ehbio.com/ImageGP/ (Chen et al., 2022).

### 2.4 Climatic data acquisition and processing

The climate data utilized in this study originated from the WorldClim 2 database (https://www.worldclim.org/data/index.html; Fick and Hijmans, 2017), released in 2020. This comprehensive dataset encompassed 19 bioclimatic variables, capturing monthly temperature and precipitation patterns from 1970 to 2000. Each data point was characterized by a spatial resolution of 30 arc-seconds (~1 km^2^). For the purpose of this analysis, ArcGIS 10.2 software was employed to import 19 vector files containing the bioclimatic variables, along with a provincial boundary map specific to Shandong Province. These climate factor vector files for Shandong Province were processed using various tools including ArcToolbox, Spatial Analyst, and extraction and analysis functions within the ArcGIS menu. During the data extraction process, latitude and longitude information for various regions were incorporated, enabling the isolation of the 19 bioclimatic variables. Subsequently, a Pearson correlation analysis was conducted using SPSS 26.0 software (IBM Corporation, Armonk, New York, USA) to assess the relationship between these variables. Climate variables with a correlation coefficient below 0.80 were deemed uncorrelated and were thus retained for further analysis.

## 3 Results

### 3.1 Species distribution and diversity in Shandong and adjacent regions

Excluding widely distributed and endemic species, the analysis identified 2410 shared species. The three most abundant species, *Amaranthus viridis* L., *Cynanchum thesioides* (Freyn) K. Schum., and *Achyranthes bidentata* Blume, accounted for a significant proportion of the total population. Notably, the top 20 abundant species, which are commonly found in at least two regions, exhibited distinct regional distribution patterns (**Supplementary Figure 1**). Species such as *Sonchus oleraceus* L., *Physalis angulata* L., and *Euphorbia maculata* L. were primarily distributed in provinces south of Shandong, including Jiangsu, Anhui, and Zhejiang. Conversely, plants like *Kali collinum* (Pall.) Akhani & Roalson, *Erodium stephanianum* Willd., and *Viola prionantha* Bunge were more prevalent in Shandong and northern provinces. The findings further revealed that 21 species had the Shandong Peninsula as their northernmost distribution boundary, while 15 species had it as their southernmost boundary. Additionally, 24 species were endemic to the Shandong Peninsula, indicating its rich endemic biodiversity (**Supplementary Table 2**). These observations collectively suggest that Shandong Province serves as an intermediary zone for species distribution.

**Figure 2:**
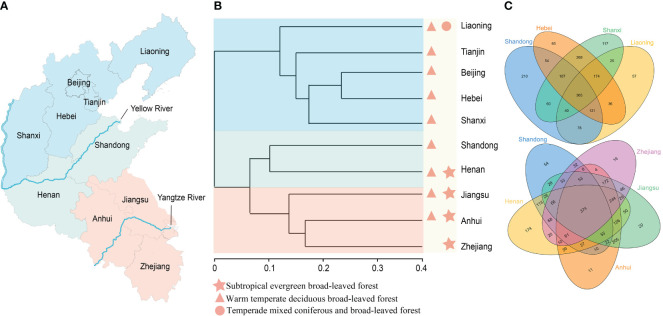
An analysis of the relationships and similarities among the Shandong Peninsula and its adjacent regions, leveraging clustering and Venn diagrams to understand species composition, particularly emphasizing the Yellow River’s barrier effect on floristic diversity. **(A)** Representation of the geographical positioning of the Yellow River within the surveyed regions. **(B)** Floristic analyses of the Shandong Peninsula and adjacent regions, revealing three primary clades delimited by the Yellow River. **(C)** Identification of shared species among distinct regions.

When comparing species diversity between Shandong and its neighboring provinces, Jiangsu, Henan, and Anhui exhibited higher species richness, contributing 64.27%, 62.03%, and 58.96% to the total 2410 species, respectively. Notably, regions south of Shandong tended to harbor greater species diversity. To visually illustrate the similarities and differences in species distribution, a Venn diagram was constructed with Shandong as the reference boundary (**Figure 2C**). The analysis revealed fewer shared species between Shandong and northern provinces compared to those with southern provinces. Northern provinces exhibited a significantly higher number of unique species (449 species) than southern provinces (275 species). Moreover, the number of shared species between Shandong and southern regions was higher than with northern provinces, indicating a closer affinity between Shandong and southern regions. Specifically, Shandong shared 795 species with Henan, 657 species with Jiangsu, and 610 species with Anhui, compared to 647 species with Hebei, 572 species with Shanxi, and 604 species with Liaoning. These findings suggest a strong similarity between the plant flora of Shandong and those of southern regions.

### 3.2 Floristic affinity based on species similarity

Based on species similarity coefficients, a hierarchical clustering tree was constructed for the ten regions, revealing distinct floristic affinities. As depicted in **Figures 2A, B**, these regions were primarily divided into three clades. The northern clade included Liaoning, Tianjin, Beijing, Hebei, and Shanxi, all situated north of the Yellow River. The southern clade comprised Jiangsu, Anhui, and Zhejiang, regions either located in the southern part of the country or intersected by the Yangtze River. The third clade grouped Shandong and Henan, both positioned along the Yellow River and exhibiting high species similarity, particularly with respect to the southern flora. The clustering tree demonstrated a gradual transition from evergreen broad-leaved forest to deciduous broad-leaved forest, followed by a shift to a mixed coniferous and broad-leaved forest. Shandong occupies a pivotal position within this transition zone, bridging the warm-temperate deciduous broad-leaved forest and the temperate mixed coniferous and broad-leaved forest.

**Figure 3:**
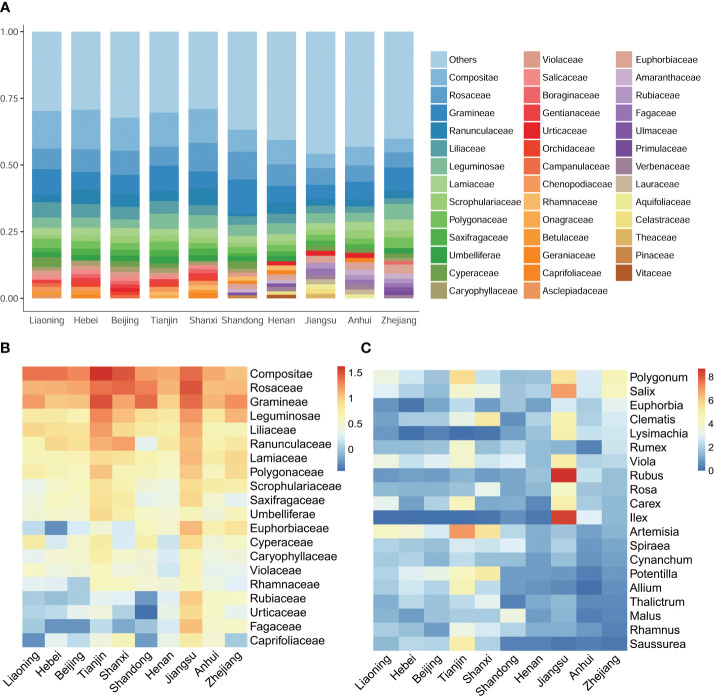
Comparative floristic analysis and contents across distinct regions. **(A)** The top 20 most abundant families and their respective percentages in each surveyed region. **(B, C)** present heatmaps that visualize the relative abundances of the top 20 families and genera, respectively. The color gradation in these heatmaps reflects the relative abundances, which have been transformed using a logarithmic scale with base 2.

To further investigate species distribution patterns, taxonomic compositions across the regions were compared. At the family level, the Compositae, Rosaceae, and Gramineae emerged as the most abundant families, collectively contributing to approximately 25% of the overall species diversity (**Figure 3B**). However, significant disparities were observed in the distribution of plant families between the northern and southern regions. Families such as Salicaceae, Gentianaceae, Orchidaceae, and Campanulaceae were more prevalent in the northern regions, while Rubiaceae, Fagaceae, Primulaceae, and Verbenaceae were more widespread in the southern regions. Larger families, including Rubiaceae, Urticaceae, Fagaceae, Caprifoliaceae, and Euphorbiaceae, exhibited distinct north-south distribution boundaries within Shandong (**Figure 3A**). Similarly, numerous genera displayed distinct distribution patterns within Shandong. Genera such as *Allium* L., *Artemisia* L., *Potentilla* L., and *Saussurea* DC. were more abundant in the northern part of Shandong, whereas genera such as *Euphorbia* L., *IIex* L. and *Lysimachia* L. were more dominant in the southern regions. Additionally, eight genera, including *Elymus* L., *Echinochloa* Beauv., and *Prunus* L., displayed greater diversity in Shandong (**Figure 3C**).

### 3.3 Ecological adaptability of floristic composition

**Figure 4:**
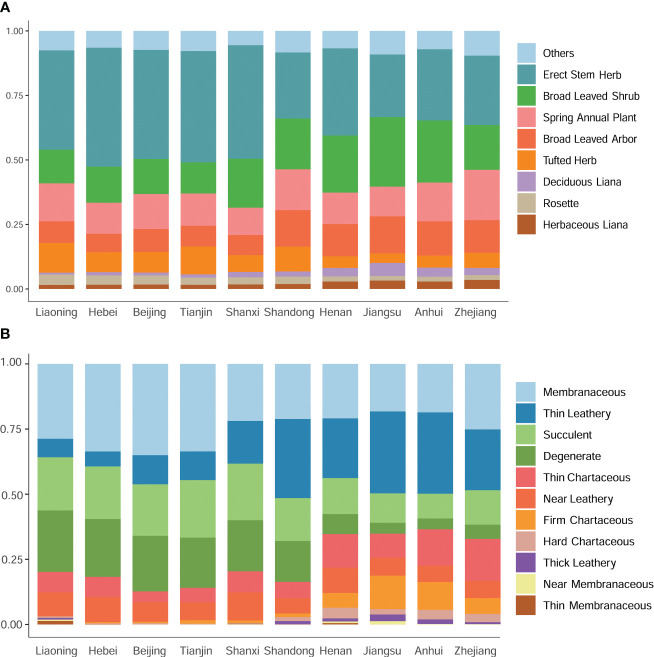
A comparative analysis of plant life forms and their associated leaf texture traits across diverse geographical regions. **(A)** Comparison of plant life forms among different regions. **(B)** Comparison of leaf texture traits among different regions.

Beyond taxonomic diversity, a marked north-south divergence in plant morphology, particularly in leaf attributes, was observed along the Shandong border. The abundance of upright-stemmed and tufted herbs was notably higher in the northern provinces compared to Shandong and its southern neighbors. Conversely, broad-leaved woody species, including both trees and shrubs, were more prevalent in Shandong and the southern regions (**Figure 4A**). In the northern reaches of Shandong, membranous, succulent, and deciduous plants dominated the foliage, whereas leathery, robust, and hard chartaceous leaves were more common in Shandong and its southern provinces (**Figure 4B**). These patterns suggest a strong association between plant distribution and ecological adaptability.

### 3.4 The special climate of Shandong peninsula

**Figure 5:**
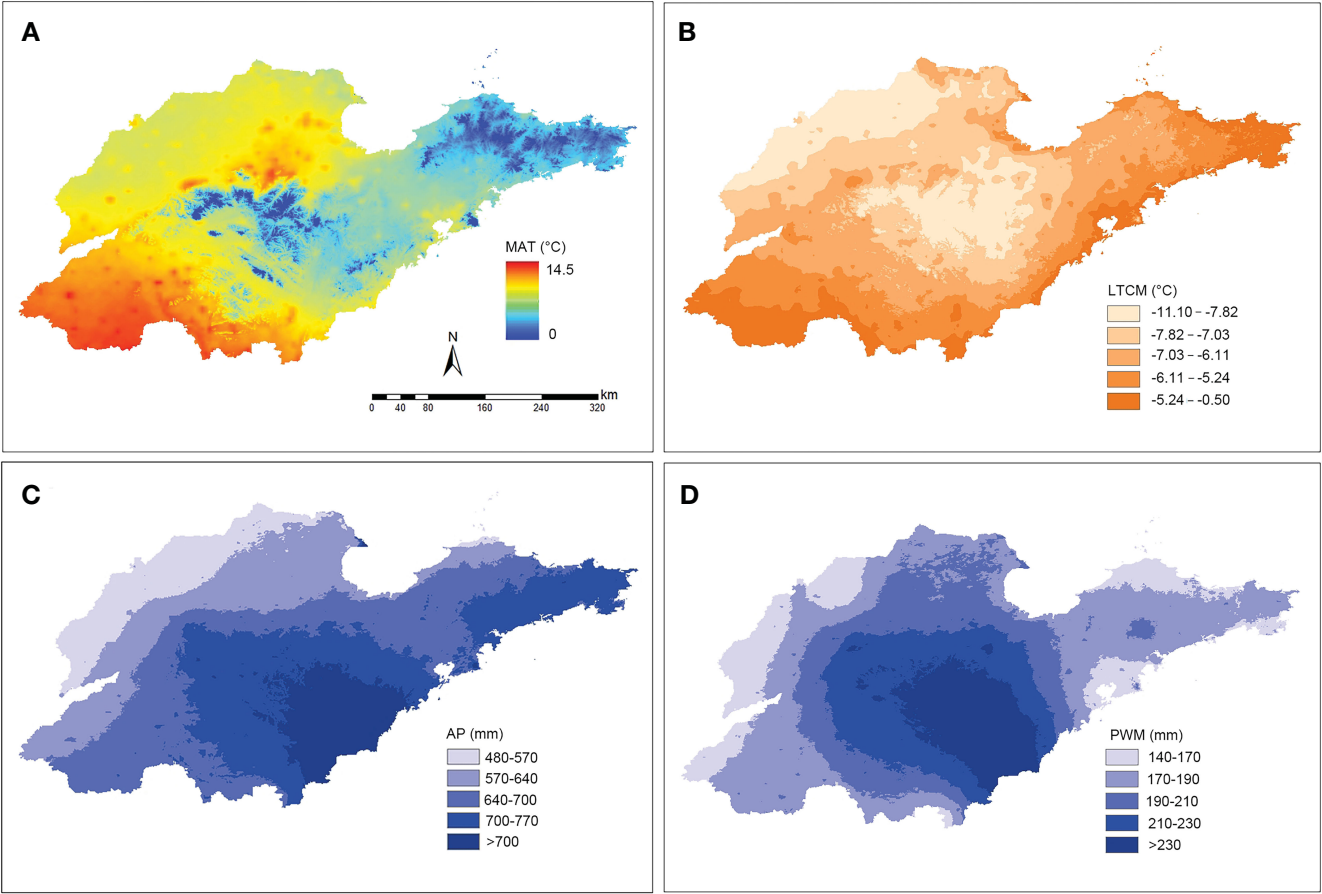
Spatial distribution of temperature and precipitation within Shandong province from 1970-2000. **(A)** Spatial visualization of mean annual temperature (MAT). **(B)** Spatial visualization of the lowest temperature in the coldest month (LTCM). **(C)** Spatial visualization of the mean annual precipitation (AP). **(D)** Spatial visualization of the mean precipitation in the wettest month (PWM).

A total of six bioclimatic variables exhibiting significant relative independence were identified across Shandong’s diverse regions. These variables included mean annual temperature, lowest temperature during the coldest season, mean temperature in the coldest season, mean annual precipitation, mean precipitation in the wettest month, and mean precipitation in the driest month. For spatial visualization, four of these variables were selected, effectively reflecting the climatic variations within the study region. During the period from 1970 to 2000, these climatic factors in the Shandong Peninsula exhibited distinct differences compared to other regions (**Figure 5**). Specifically, the average temperature was lower, while the temperatures during the coldest months were higher compared to other regions. Additionally, the annual rainfall in this region was higher than in other regions. This unique combination of a warm and humid climate favors the winter habitation and dispersal of southern plants.

## 4 Discussion

To effectively conserve biodiversity and optimize agricultural practices, a comprehensive understanding of the floristic composition and spatial patterns of biodiversity is essential (Ferrier, 2002). This study provides a detailed analysis of the plant composition in Shandong, elucidating its relationships with the floral communities of adjacent regions (**Figure 2C**). **Figure 3** highlights the distribution patterns of various taxa, revealing significant regional disparities. For instance, the species diversity of subtropical families such as Rubiaceae, Fagaceae, and Primulaceae shows a marked decline in Shandong and the northern regions, indicating that Shandong represents the northernmost limit for the diversity of these families. An extensive study was conducted on 1045 species in the Shandong Peninsula (**Supplementary Table 3**). Among these, 21 species have their northernmost distribution boundary in the Shandong Peninsula, while 15 species have their southernmost boundary there (**Supplementary Table 2**). These findings suggest that the Shandong Peninsula serves as a crucial transition zone for floristic elements between southern and northern China.

In recent years, numerous studies have demonstrated that plant species distribution is frequently influenced by specific environmental and climatic factors (Haerdtle et al., 2006; Zelnik and Carni, 2013; Farooq et al., 2019; Abdelaal et al., 2020; Zeb et al., 2021). Consistent with these studies, this investigation revealed regional disparities in plant functional traits in eastern China, particularly evident in leaf characteristics, which are highly responsive to environmental changes. These disparities follow a north-south axis, with Shandong serving as the dividing line (**Figure 4**). Geographically situated between the Yellow Sea and the Bohai Sea, the climate of the Shandong Peninsula is profoundly shaped by the surrounding marine environment. This influence is reflected in the peninsula’s distinct climatic characteristics, which, despite its northern geographical location, resemble those of southern regions. Specifically, the Shandong Peninsula exhibits temperature seasonality, annual temperature range, and precipitation seasonality patterns that align more closely with southern regions (**Figure 5**). Consequently, this environment is conducive to the natural growth of certain southern species, including *Camellia sinensis* (L.) Kuntze, *C. japonia* L., *Glochidion puberum* (L.) Hutch., and *M. thunbergii*, which often dominate the evergreen shrub communities.

The Shandong Peninsula is further distinguished by the presence of the Laoshan Mountain ridge, which extends from northeast to southwest. The ridge main peak reaches an elevation to 1132 meters, creating a natural barrier that alters air flow and forms distinct climatic zones on either side (Fan and Hu, 2003). During winter, the cold air from the north is obstructed by the mountains, resulting in markedly different climates on the northern and southern slopes. The northern and northwestern slopes experience cooler winters and higher rainfall, establishing a natural southern limit for the distribution of many plant species. The flora of this region reflects its northeastern Chinese affinities, with species such as *Anemone raddeana* Regel, *M. amurensis*, *Tilia amurensis* Rupr., and *Pinus koraiensis* Siebold & Zucc. being characteristic. These unique climatic factors contribute to the diverse species assemblage observed in the Shandong Peninsula, which exhibits a distinct north-south floristic composition.

Throughout geological history, the Shandong Peninsula was once connected to both the Liaodong Peninsula and Japan. However, at the end of the Miocene era of the Tertiary period, the connection between Japan and the Asian continent ruptured. Subsequently, at the commencement of the Quaternary period, the Liaodong Peninsula separated from the Shandong Peninsula (Jin and Yu, 1982). This historical disjunction is evidenced by the presence of endemic plant species. For instance, *Pterocarya rhoifolia* Siebold & Zucc., is exclusively found in Shandong and Japan, while *Vaccinium oldhamii* Miq. and *Pinus densiflora* Siebold & Zucc. are endemic to Shandong, Liaoning, and Japan. Similarly, *Primula loeseneri* Kitag., *Atractylodes koreana* (Nakai) Kitam, *Rhamnus koraiensis* C. K. Schneid., *Carlesia sinensis* Dunn, and *Trigonotis radicans* (Turcz.) Stev. are restricted to Shandong and Liaoning. These floristic patterns provide convincing evidence of ancient geographical shifts.

An analysis focusing on the floristic assessment within the Yellow River basin, encompassing Shandong, Shanxi and eight additional provinces, reveals a distinct pattern of species distribution. This pattern is consistent with the hypothesis that the Yellow River acts as a significant riverine barrier to plant migration and distribution. The riverine barrier hypothesis, first proposed by Alfred Russell Wallace in the mid-nineteenth century (Wallace, 1852), has been foundational in understanding biodiversity patterns and species distribution in regions such as the Amazon basin (Gascon et al., 2000; Caze et al., 2016). It posits those large rivers can serve as natural barriers to the organism dispersal, leading to genetic divergence and the formation of distinct biological communities on either side (Nazareno et al., 2017; Musher et al., 2022). The Yellow River, as the third longest river in Asia and a major geographical feature of China, provides an excellent case study for this hypothesis. The unique flora of Shandong, when compared to both Liaoning and southern provinces, can be partially attributed to the Yellow River’s barrier effect. This river, with its massive sediment load and depositional processes, has significantly shaped the landscape and ecological niches of the region. The Yellow River Delta, formed by sediment deposition from the Loess Plateau, creates a natural barrier to plant migration in northwest Shandong (Yang et al., 2003; He et al., 2017). This barrier, along with the river’s physical and ecological characteristics, likely plays a crucial role in determining plant species distribution and diversity in the region. Numerous studies have highlighted the impact of the Yellow River and its delta on local genetic diversity and species distribution (Liang et al., 2019; Liu et al., 2021; Wu et al., 2022).

In addition to its impact on species distribution, the Yellow River’s riverine barrier has significant implications for conservation efforts. Understanding how these barriers shape biodiversity patterns can aid conservationists in identifying critical habitats and prioritize areas for protection (Ren et al., 2017; Li et al., 2019). For instance, recognizing the Yellow River acts as a barrier to plant migration can inform strategies for replanting and restoration efforts, ensuring that native species are appropriately distributed and possess the necessary genetic diversity to thrive. This knowledge can be pivotal in maintaining ecological balance and promoting the resilience of plant communities in the region.

In summary, this study provides a comprehensive examination of the floristic composition of the Shandong Peninsula, highlighting its pivotal role as a transition zone for plant distribution between southern and northern China. These findings support the riverine barrier hypothesis and underscores the crucial role of the Yellow River in shaping the patterns of regional plant distribution across the North-South divide in China.

## Data Availability

The original contributions presented in the study are included in the article/**Supplementary Material**, further inquiries can be directed to the corresponding author/s.
