# Supplementary material for: Floristic analyses of Shandong peninsula and adjacent areas indicate the barrier effect of the Yellow river on floristic diversity
Source: Front Plant Sci. 2024 Aug 15;15:1419876. doi: 10.3389/fpls.2024.1419876 (PMC11358103; doi:10.3389/fpls.2024.1419876)
Supplement: Supplementary file 2 [file Table2.docx]

Supplementary Material

Supplementary Table 2. The taxon distributed with the Shandong Peninsula as the boundary

| Family | Species | Northernmost boundary | Southernmost boundary | Endemic species |
| --- | --- | --- | --- | --- |
| [Amaranthaceae](https://www.iplant.cn/info/Amaranthaceae) | *Amaranthus polygonoides* |  |  | √ |
|  | *Lycoris squamigera* | √ |  |  |
| Apocynaceae | *Trachelospermum jasminoides* | √ |  |  |
| [Betulaceae](https://www.iplant.cn/info/Betulaceae) | *Carpinus mengshanensis* |  |  | √ |
| [Boraginaceae](https://www.iplant.cn/info/Boraginaceae) | *Trigonotis radicans* |  | √ |  |
|  | *Trigonotis tenera* |  |  | √ |
| [Compositae](https://www.iplant.cn/info/Compositae) | *Atractylodes koreana* |  | √ |  |
| [Crassulaceae](https://www.iplant.cn/info/Crassulaceae) | *Phedimus floriferus* |  |  | √ |
|  | *Sedum uniflorum*var.*japonicum* |  |  | √ |
| [Cyperaceae](https://www.iplant.cn/info/Cyperaceae) | *Carex jiaodongensis* |  |  | √ |
|  | *Carex wahuensis*subsp*. robusta* |  |  | √ |
| Elaeagnaceae | *Elaeagnus macrophylla* | √ |  |  |
| [Eriocaulaceae](https://www.iplant.cn/info/Eriocaulaceae) | *Eriocaulon taishanense* | √ |  |  |
| Euphorbiaceae | *Glochidion puberum* | √ |  |  |
|  | *Sapium japonicum* | √ |  |  |
| Geraniaceae | *Geranium koreanum* |  |  | √ |
|  | *Agrostis infirma* |  |  | √ |
|  | *Zoysia macrostachya* | √ |  |  |
| [Juglandaceae](https://www.iplant.cn/info/Juglandaceae) | *Pterocarya rhoifolia* |  |  | √ |
| [Lamiaceae](https://www.iplant.cn/info/Lamiaceae) | *Salvia weihaiensis* |  |  | √ |
| [Lauraceae](https://www.iplant.cn/info/Lauraceae) | *Lindera erythrocarpa* | √ |  |  |
|  | *Lindera glauca* | √ |  |  |
|  | *Machilus thunbergii* | √ |  |  |
| [Leguminosae](https://www.iplant.cn/info/Leguminosae) | *Maackia amurensis* |  | √ |  |
|  | *Vicia kulingana* | √ |  |  |
| [Liliaceae](https://www.iplant.cn/info/Liliaceae) | *Allium taishanense* |  |  | √ |
|  | *Lilium tsingtauense* |  |  | √ |
|  | *Liriope spicata* var*. humilis* |  |  | √ |
|  | *Smilax china* | √ |  |  |
| Molluginaceae | *Mollugo verticillata* | √ |  |  |
| Orchidaceae | *Cleisostoma scolopendrifolium* | √ |  |  |
| Primulaceae | *Primula jesoana* |  | √ |  |
|  | *Primula loeseneri* |  | √ |  |
| Ranunculaceae | *Anemone raddeana* |  | √ |  |
|  | *Anemone shikokiana* |  |  | √ |
|  | *Caltha palustris*var.*sibirica* |  | √ |  |
|  | *Clematis fusca* |  | √ |  |
|  | *Clematis patens* |  | √ |  |
|  | *Delphinium grandiflorum* |  |  | √ |
|  | *Clematis hexapetala* |  |  | √ |
| Rhamnaceae | *Rhamnus koraiensis* |  | √ |  |
|  | *Rhamnus laoshanensis* |  |  | √ |
| [Rosaceae](https://www.iplant.cn/info/Rosaceae) | *Pyrus trilocularis* |  |  | √ |
|  | *Rosa maximowicziana* |  | √ |  |
| [Rubiaceae](https://www.iplant.cn/info/Rubiaceae) | [*Spermacoce shandongensis*](https://www.iplant.cn/info/Spermacoce%20shandongensis) |  |  | √ |
| Rutaceae | *Zanthoxylum armatum* | √ |  |  |
| Sabiaceae | *Meliosma myriantha* | √ |  |  |
|  | *Meliosma oldhamii* | √ |  |  |
|  | *Salix luzhongensis* |  |  | √ |
| [Scrophulariaceae](https://www.iplant.cn/info/Scrophulariaceae) | *Torenia taishanensis* |  |  | √ |
| [Stemonaceae](https://www.iplant.cn/info/Stemonaceae) | *Stemona shandongensis* | √ |  |  |
| Symplocaceae | *Symplocos chinensis* | √ |  |  |
| Theaceae | *Camellia japonica* | √ |  |  |
| Tiliaceae | *Tilia amurensis* |  | √ |  |
|  | *Tilia amurensis* |  | √ |  |
|  | *Tilia jiaodongensis* |  |  | √ |
|  | *Tilia taishanensis* |  |  | √ |
| [Umbelliferae](https://www.iplant.cn/info/Umbelliferae) | *Carlesia sinensis* |  | √ |  |
|  | *Ligusticum ajanense* |  | √ |  |
| [Verbenaceae](https://www.iplant.cn/info/Verbenaceae) | *Vitex negundo* | √ |  |  |
