# Supplementary material for: Floristic analyses of Shandong peninsula and adjacent areas indicate the barrier effect of the Yellow river on floristic diversity
Source: Front Plant Sci. 2024 Aug 15;15:1419876. doi: 10.3389/fpls.2024.1419876 (PMC11358103; doi:10.3389/fpls.2024.1419876)
Supplement: Supplementary file 4 [file Image1.pdf]

Supplementary Material

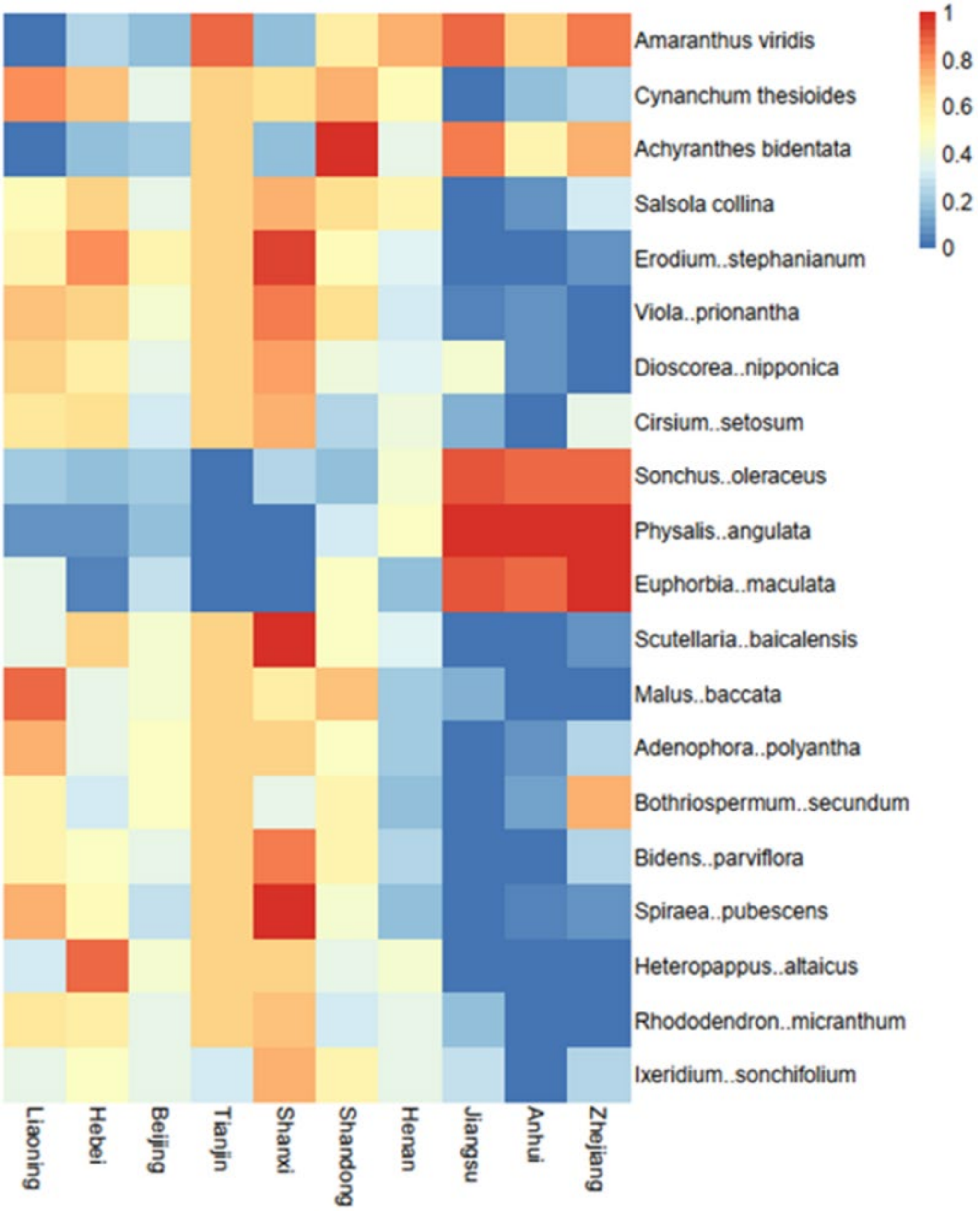

Supplementary Figure 1. A heatmap illustrating the distribution and relative abundance of the most commonly shared species in at least two, but not all, regions. The color grades indicate their relative abundances which transformed by the log base 10.
